# Supplementary figures and images for: Long Non-Coding RNA Expression Levels Modulate Cell-Type-Specific Splicing Patterns by Altering Their Interaction Landscape with RNA-Binding Proteins
Source: Genes (Basel). 2019 Aug 6;10(8):593. doi: 10.3390/genes10080593 (PMC6722645; doi:10.3390/genes10080593)

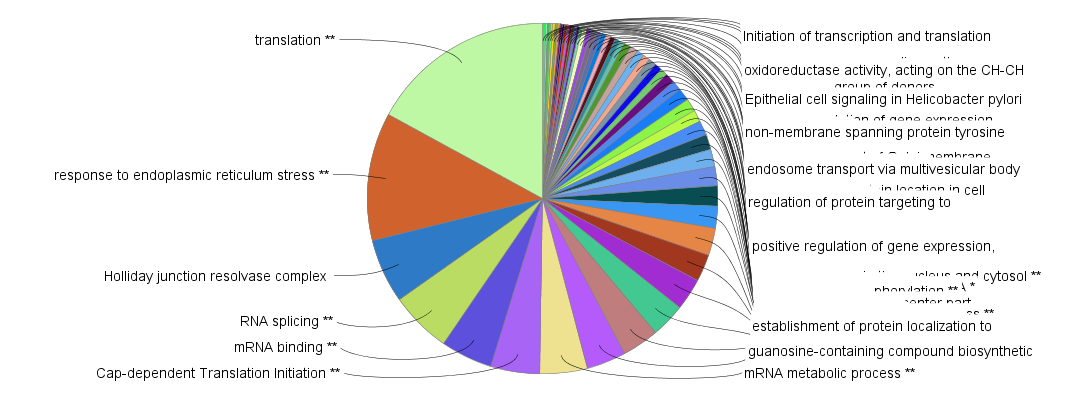

Supplement: Supplementary file 1 [file genes-10-00593-s001.zip › Supplementary materials/Supplementary material 10.png]

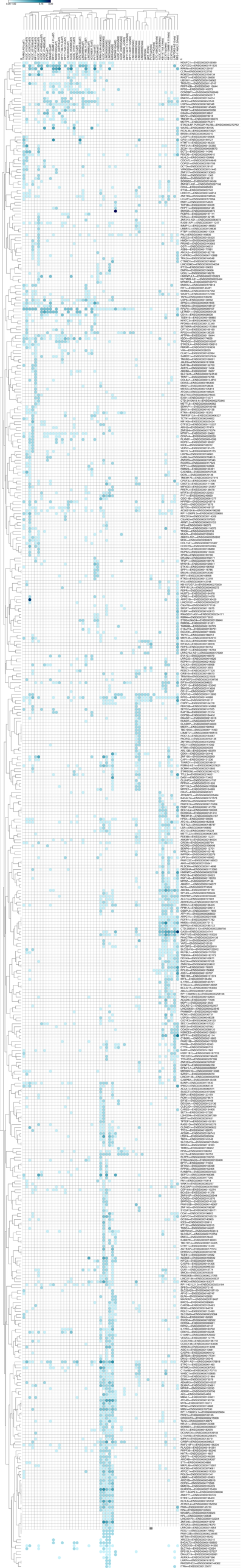

Supplement: Supplementary file 1 [file genes-10-00593-s001.zip › Supplementary materials/Supplementary material 4.pdf]

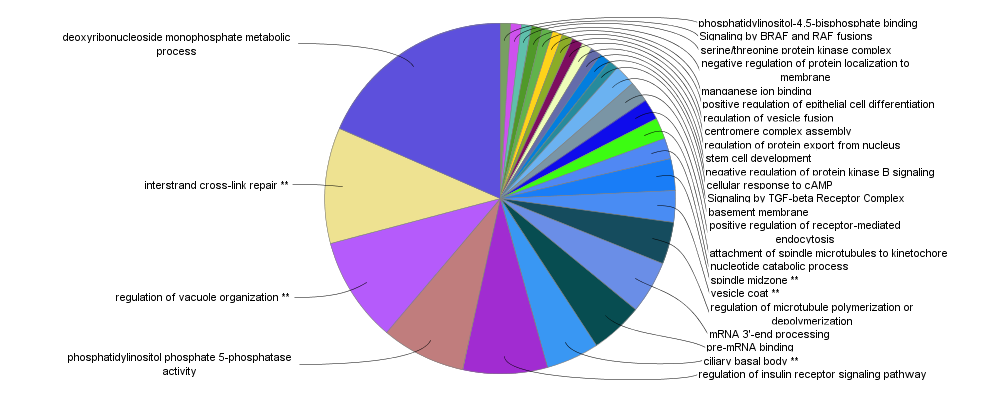

Supplement: Supplementary file 1 [file genes-10-00593-s001.zip › Supplementary materials/Supplementary material 8.png]

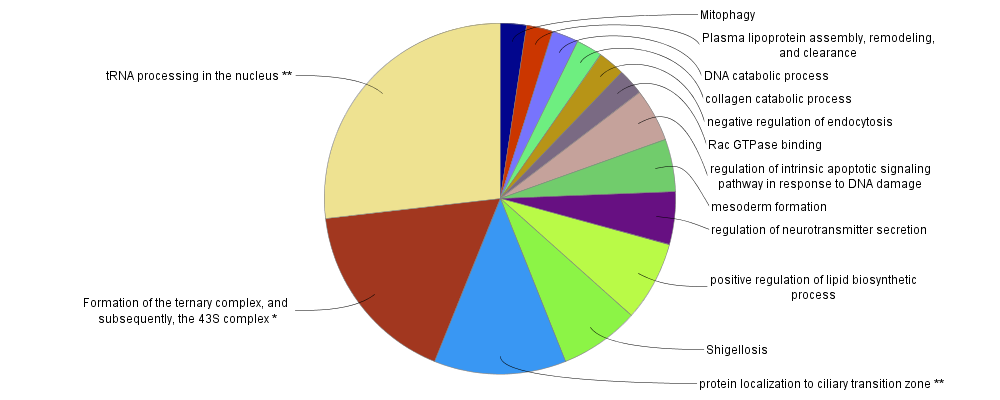

Supplement: Supplementary file 1 [file genes-10-00593-s001.zip › Supplementary materials/Supplementary material 6.png]

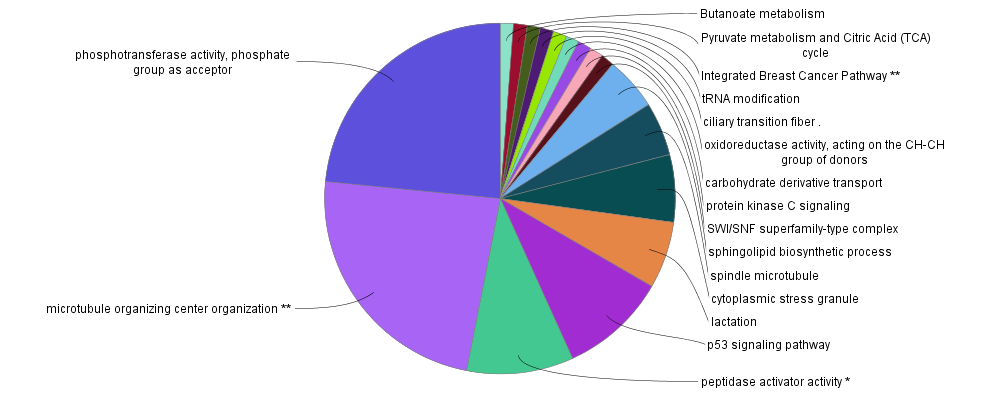

Supplement: Supplementary file 1 [file genes-10-00593-s001.zip › Supplementary materials/Supplementary material 7.png]

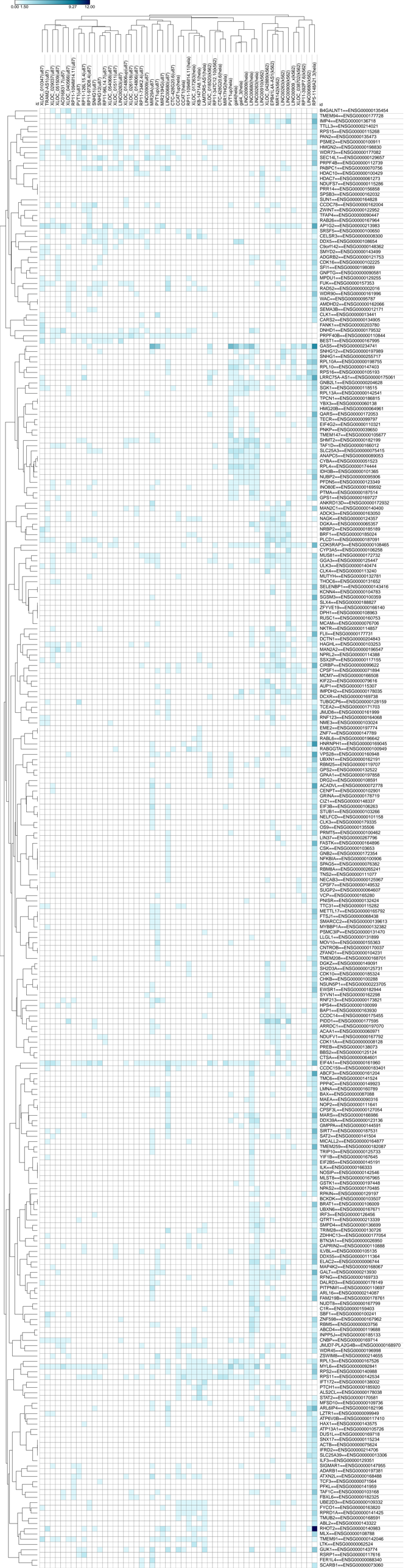

Supplement: Supplementary file 1 [file genes-10-00593-s001.zip › Supplementary materials/Supplementary material 5.pdf]

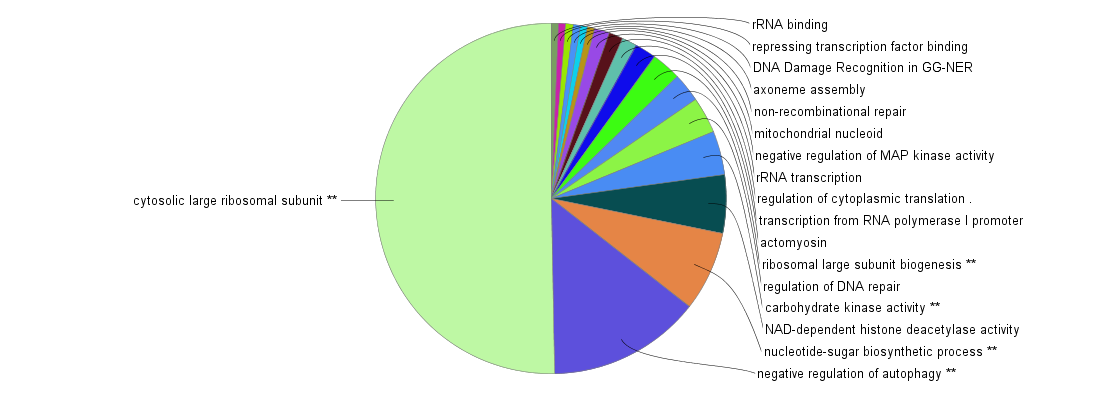

Supplement: Supplementary file 1 [file genes-10-00593-s001.zip › Supplementary materials/Supplementary material 9.png]
